# Necrotizing fasciitis—A catastrophic complication following routine tibia fracture surgery: A case report and literature review: Erratum

**DOI:** 10.1097/MD.0000000000007360

**Published:** 2017-06-23

In the article, “Necrotizing fasciitisA catastrophic complication following routine tibia fracture surgery: A case report and literature review”,^[1]^ which appeared in Volume 96, Issue 23 of *Medicine*, the caption of Figure 1 should have appeared as “Postoperative radiograph of tibia fracture MIPO surgery showing good reduction and minimal incision. MIPO  =  minimally invasive plate osteosynthesis.”
